# Targeted PLK1 suppression through RNA interference mediated by high‐fidelity Cas13d mitigates osteosarcoma progression via TGF‐β/Smad3 signalling

**DOI:** 10.1111/jcmm.18400

**Published:** 2024-05-23

**Authors:** Yi Yuan, Daigui Cao, Anwei Zhang, Zhiwei Liu, Zhongliang Deng, Shengli Zhang

**Affiliations:** ^1^ Department of Orthopedics Chongqing General Hospital Chongqing China; ^2^ Department of Orthopedics, The Second Affiliated Hospital Chongqing Medical University Chongqing China; ^3^ Department of Orthopedics Dazhou Second People's Hospital of Sichuan Province Dazhou China

**Keywords:** hfCas13d, osteosarcoma, PLK1, Smad3

## Abstract

Osteosarcoma is the most common primary bone malignancy in children and adolescents. Overexpression of polo‐like kinase 1 (PLK1) is frequent in osteosarcoma and drives disease progression and metastasis, making it a promising therapeutic target. In this study, we explored PLK1 knockdown in osteosarcoma cells using RNA interference mediated by high‐fidelity Cas13d (hfCas13d). PLK1 was found to be significantly upregulated in osteosarcoma tumour tissues compared to normal bone. sgRNA‐mediated PLK1 suppression via hfCas13d transfection inhibited osteosarcoma cell proliferation, induced G2/M cell cycle arrest, promoted apoptosis, reduced cell invasion and increased expression of the epithelial marker E‐cadherin. Proximity labelling by TurboID coupled with co‐immunoprecipitation identified novel PLK1 interactions with Smad3, a key intracellular transducer of TGF‐β signalling. PLK1 knockdown impaired Smad2/3 phosphorylation and modulated TGF‐β/Smad3 pathway inactivation. Finally, in vivo delivery of hfCas13d vectors targeting PLK1 substantially attenuated osteosarcoma xenograft growth in nude mice. Taken together, this study highlights PLK1 as a potential therapeutic target and driver of disease progression in osteosarcoma. It also demonstrates the utility of hfCas13d‐mediated gene knockdown as a strategy for targeted therapy. Further optimization of PLK1 suppression approaches may ultimately improve clinical outcomes for osteosarcoma patients.

## 1 INTRODUCTION

Osteosarcoma (OS), the most prevalent primary malignant bone tumor affecting children and young adults, is a type of bone cancer that typically originates in the long bones of the body such as the arms or legs. Current standard treatment involves neoadjuvant chemotherapy, surgical resection of the tumour and adjuvant chemotherapy. However, patient outcomes have remained relatively stagnant over the past 30 years, with 5‐year overall survival around 60%–70% for patients with localized tumours at diagnosis. Survival dramatically decreases to about 20% for those initially presenting with metastasis.^1^ Approximately 10% of patients have detectable distant metastases at diagnosis, but eventually almost 50% of patients develop metastases, which commonly lead to mortality.^2^ The aggressive and highly metastatic nature of OS underscores the urgent need for more effective therapeutic strategies aimed at improving clinical outcomes.

Polo‐like kinase 1 (PLK1) is a serine/threonine kinase that plays critical roles in cell cycle progression, particularly in the G2/M checkpoint, mitosis and cytokinesis.^3^, ^4^, ^5^ PLK1 expression and activity are tightly regulated during cell cycle progression. Aberrant overexpression of PLK1 has been observed in many cancer types and often correlates with poor prognosis.^6^, ^7^, ^8^ Overexpression of PLK1 has been observed in various cancers including lung cancer, where it promotes metastasis through mechanisms that are not fully understood.^9^ Jang et al.^9^ demonstrated that PLK1 directly phosphorylates vimentin at residues S339, T327 and S83 which activates TGF‐beta/Smad signalling and promotes nuclear translocation of Smad2/3 to induce expression of the immune checkpoint protein PD‐L1, resulting in metastatic phenotypes in vitro and in vivo. Due to its oncogenic functions, PLK1 has emerged as a promising target for cancer therapy.^10^ Small molecule inhibitors targeting the kinase domain or polo‐box domain of PLK1 have been developed and several have progressed to clinical trials, including BI2536, BI6727, NMS‐1286937, volasertib and GSK461364.^10^, ^11^, ^12^ Liang group kinome‐wide clustered regularly interspaced short palindromic repeat (CRISPR)‐associated protein (Cas) 9 knockout screenings have identified PLK1 as a critical kinase for the survival and proliferation of human osteosarcoma cells, offering new insights and potential therapeutic avenues for this aggressive cancer.^12^ These inhibitors target the kinase domain of PLK1 and prevent its enzymatic activity, leading to disrupted cell division and apoptosis in cancer cells.^11^ However, there are still challenges with PLK1 inhibitors that need to be addressed. The inhibitors often lack specificity among PLK family members and can cause side effects. Cancer cells may also develop resistance by mutating the PLK1 kinase domain. Overall, PLK1 remains a promising target but creative approaches are still required to achieve robust clinical responses. Recent evidence suggests PLK1 may contribute to metastasis and immune evasion in lung adenocarcinoma through phosphorylation of the intermediate filament protein vimentin.

CRISPR‐Cas13 systems have emerged as promising tools for targeted RNA degradation and gene therapy applications.^13^, ^14^, ^15^ However, the wild‐type Cas13 proteins exhibit substantial collateral RNA cleavage activity, limiting their therapeutic potential. Recently, high‐fidelity Cas13d (hfCas13d) variants were engineered through mutagenesis to eliminate the collateral effects while maintaining robust on‐target RNA cleavage activity.^16^ Compared to wild‐type Cas13d, hfCas13d variants exhibited high specificity in degrading target RNAs with no detectable transcriptome‐wide off‐target effects in mammalian cells. Additionally, hfCas13d‐mediated gene knockdown did not affect cell viability or induce toxicity in vivo.^16^ These hfCas13d systems overcome a major obstacle for therapeutic applications of RNA‐targeting. The high specificity of hfCas13d makes it a promising platform for RNA‐targeting gene therapies. Preclinical studies have shown that hfCas13d can efficiently knock down disease‐causing transcripts in vivo without adverse effects.^17^

In the present study, we first explored the effects of hfCas13d‐mediated PLK1 knockdown on osteosarcoma cell viability, apoptosis, proliferation and invasion to elucidate the role of PLK1 in osteosarcoma cells. Furthermore, we employed a TurboID‐mediated proximity labelling strategy to map the PLK1 protein and protein interaction (PPI) network. Through immunoprecipitation assays, we further confirmed that PLK1 could interact with Smad3 to consequently influence TGF‐beta/Smad signalling pathway activation. Finally, we constructed a subcutaneous xenograft model in vivo to evaluate the potential of hfCas13d‐mediated PLK1 knockdown delivered by lentiviral vectors as a therapeutic strategy for osteosarcoma. Our findings highlight PLK1 as a potential therapeutic target in osteosarcoma and demonstrate the utility of CRISPR‐Cas13d technology for targeted gene suppression. Further research could optimize Cas13d‐mediated PLK1 targeting and explore combinatorial strategies to improve treatment efficacy.

## 2 MATERIALS AND METHODS

### 2.1 Plasmid construction and cloning

The plasmid containing hfCas13d (NLS‐hfCas13d‐NLS) and U6 promoter driving the direct repeat sequence of gRNA was obtained from Addgene (catalogue 190034). Furthermore, the sequences of hfCas13d and U6 promoter driving the direct repeat sequence of gRNA were amplified using PCR polymerase (cat# P505, Vazyme, China) and then seamlessly cloned into pLenti series vector with puromycin screening marker. To generate a target‐specific knockdown sequence for PLK1 (Homo sapiens), we designed the guide RNA (sgRNA) using the online tool available at https://cas13design.nygenome.org/. The resulting sgRNA sequences were listed as follows: sgRNA1: 5′‐CTATCTGTCGCAGGTAGTAGCGGGCCTCAG‐3′; sgRNA2: 5′‐CGTGGGTCCACTAGGACCTCCGGAATTTCT‐3′; sgRNA3:5′‐AGCAGCAAAGACTTAGGCACGATCTTGCCT‐3′; sgRNA‐control: 5′‐CGTCTGGCCTTCCTGTAGCCAGCTTTCATC‐3′. The resulting sgRNA sequences were inserted into the BbsI restriction site. Sanger sequencing was performed by Sangon (China) to validate all the aforementioned sequences.

### 2.2 Cell culturing

The 293FT cell line was acquired from Thermofisher (catalogue #R700‐07) and maintained in Dulbecco's modified Eagle's medium (DMEM, catalogue #SH30243.01, Gibco) supplemented with 10% fetal bovine serum (FBS) (catalogue #A511‐001, Lonsera). MG‐63 and U2OS osteosarcoma cell lines were procured from Procell and cultured in minimal essential medium (MEM, Procell, China) or McCoy's 5A medium (Procell, China), respectively, with 10% FBS supplementation. All cell lines were preserved under standard conditions of 37°C and 5% CO_2_. Upon reaching approximately 90% confluence, cells were passaged.

### 2.3 Lentivirus packaging and transfection

To generate lentiviral particles, the packaging plasmid psPAX2, envelope plasmid pMD2.G and lentiviral transfer vectors were co‐transfected into 293FT cells at a ratio of 3:2:4 utilizing PEI 40000 (catalogue 40816ES02, Yeasen, China) at a concentration of 2 mg/mL following the manufacturers' instructions. At 48–72 h post‐transfection, viral supernatants were harvested and purified through 0.45 μm filters. MG‐63 and U2OS cells were subsequently transduced with lentivirus and stable cells were obtained through supplementation with 2 μg/mL puromycin (catalogue ST551, Beyotime, China).

### 2.4 RNA extraction and quantitative real‐time PCR

Prior to RNA extraction, cells were washed once with 1X pre‐cool phosphate‐buffered saline (PBS). Total cellular RNA was isolated using TRNzol reagent (Tiangen) following the manufacturer's recommendations and RNA concentrations were quantified by spectrophotometry (NanoDrop 3000, USA). Reverse transcription of RNA to cDNA was performed with the RNA using GoScript™ Reverse Transcriptase (catalogue A2791, Promega, USA) followed by detection using iTaq Universal SYBR Green Supermix (catalogue 1725121, Bio‐Rad, USA) on a LightCycler 480 instrument (Roche, Germany). The mRNA expression levels were normalized to the housekeeping gene GAPDH and relative quantification was determined by the ΔΔCt method. The detailed primers (5′–3′) used for quantitative real‐time PCR are listed in Table 1.

**TABLE 1:** The sequence of primers for quantitative real‐time PCR.

Gene	Sequence
PLK1	
PLK1‐F	AAAGAGATCCCGGAGGTCCTA
PLK1‐R	GGCTGCGGTGAATGGATATTTC
MMP2	
MMP2‐F	TACAGGATCATTGGCTACACACC
MMP2‐R	GGTCACATCGCTCCAGACT
E‐cadherin	
E‐cadherin‐F	CGAGAGCTACACGTTCACGG
E‐cadherin‐R	GGGTGTCGAGGGAAAAATAGG

### 2.5 Western blot analysis and co‐immunoprecipitation assay

Immunoblotting was undertaken as previously described.^18^ In summary, whole cell extracts prepared in RIPA lysis buffer (catalogue P0013B, Beyotime, China) were separated by gel electrophoresis using polyacrylamide gels containing sodium dodecyl sulphate. Following electrophoretic separation, proteins were transferred to polyvinylidene fluoride membranes (Millipore, USA) and blocked utilizing 5% skim milk protein. Target proteins were detected by incubating membranes sequentially with specific primary antibodies and horseradish peroxidase‐linked secondary antibodies. Finally, protein bands were visualized using the ChemiScope 600 EXp imaging system (ClinX, China) and densitometry analysis was conducted using ImageJ software to quantify relative grey values.

For co‐immunoprecipitation (Co‐IP) assay, cell lysates were generated by lysing cells in lysis buffer composed of 25 mM Tris–HCl pH 7.4, 150 mM NaCl, 1% NP‐40, 1 mM EDTA and 5% glycerol. Lysates were incubated with anti‐HA antibody (catalogue 51064‐2‐AP, Proteintech, China) or anti‐Flag antibody (catalogue 20543‐1‐AP, Proteintech, China) bound to Protein A/G Magnetic Beads (catalogue HY‐K0202, MCE, USA). Immunocomplexes on beads were washed three times with lysis buffer before elution and subsequent immunoblotting analysis.

### 2.6 Colony‐forming assay

Cells were seeded at a density of 1.0 × 10^3^ cells per well in 6‐well plates and incubated for 2–3 weeks with regular media changes. Subsequently, colonies were fixed with ice‐cold methanol and stained with 1% crystal violet. Visible colonies were manually enumerated and imaged using brightfield microscopy.

### 2.7 Cell proliferation

To assess cell viability, 5.0 × 10^3^ cells were seeded per well in 96‐well plates and allowed to incubate for 24 h. Thereafter, 10 μL of CCK‐8 reagent (catalogue HY‐K0301, MCE, USA) was added to each well and incubated for 1–4 h as per the manufacturer's instructions. Absorbance was quantified at 450 nm using a microplate reader (VT, Biotek, USA).

### 2.8 Flow cytometry for apoptosis and cell cycle

Apoptosis was analysed by flow cytometry using the Annexin‐V FITC/propidium iodide (PI) apoptosis detection kit (catalogue C1062, Beyotime, China). Following digestion by 0.25% trypsin without EDTA, MG‐63 and U2OS cells were harvested, washed twice with pre‐cold PBS, and resuspended in binding buffer containing Annexin‐V FITC and PI. Cells were incubated for 15 min at room temperature protected from light. Stained cells were then analysed by flow cytometry on a Canto II instrument (BD Biosciences, USA).

Cell cycle analysis was performed by ethanol fixation and PI staining. Briefly, following experimental treatments, cells were harvested through trypsinization, washed twice with PBS and fixed in 70% ethanol overnight at −20°C. Subsequently, cells were washed twice with cold PBS, and stained with PI and RNase in the dark for 30 min at room temperature. Stained cells were analysed by flow cytometry to determine DNA content and cell cycle distribution.

### 2.9 EdU staining

Cell proliferation was assessed by 5‐ethynyl‐2′‐deoxyuridine (EdU) incorporation using the EdU detection kit (catalogue C10310‐1, RiboBio, China) per the manufacturer's protocol. In brief, following experimental treatments, cells were incubated with medium containing 50 μM EdU for 2 h prior to fixation in 4% paraformaldehyde. EdU labelling was performed according to kit instructions and nuclei were counterstained with Hoechst 33342. Fluorescent detection and quantification of EdU‐positive cells was carried out by flow cytometry and fluorescent imaging was conducted utilizing an inverted epifluorescence microscope (A1R‐PLUS, Nikon, Japan).

### 2.10 Biotin labelling with TurboID and mass spectrometry analysis

The assay was performed as described previously.^19^ Cells stably expressing TurboID‐PLK1 were generated by lentiviral transduction. Initially, TurboID‐PLK1 expressing cells were seeded in 15 cm dishes overnight. The following day, 2 μg/mL doxycycline was added to the complete media for 24 h to induce TurboID‐PLK1 expression. Subsequently, media was replaced with 50 μM biotin‐containing media and incubated for 30 min at 37°C. The labelling reaction was terminated by washing three times with cold PBS. Cells were collected by scraping and centrifugation at 1500*g* for 10 min at 4°C. Cell pellets were lysed in RIPA buffer for 30 min at 4°C, followed by centrifugation at 15,000*g* for 30 min to isolate total proteins. Biotinylated proteins were enriched using streptavidin beads (catalogue P2151, Beyotime, China) by overnight incubation at 4°C. The beads were then washed sequentially with RIPA buffer, 1 M KCl, 0.1 M NaHCO_3_ and 2 M urea to remove non‐specifically bound proteins. Finally, enriched proteins were eluted by boiling bead samples in 50 μL SDT buffer (4% SDS, 100 mM Tris, 1 mM DTT, pH = 7.6) for 20 min at 95°C. Following magnetic bead separation, the enriched protein eluates were submitted to APTBIO for subsequent proteomic analysis by MS detection.

### 2.11 Animals

Xenograft tumours were established by subcutaneous injection of human cancer cells into immunodeficient mice. Specifically, 1 × 10^6^ MG‐63 cells suspended in 100 μL PBS were injected into the subcutaneous of male BALB/c nude mice (Dashuo Biotech, China) maintained under specific pathogen‐free conditions. Approximately 7 days after tumour inoculation, concentrated lentiviral vectors (10^8^ TU/mL, 100 μL volume) encoding hfCas13d and therapeutic sgRNAs were intratumorally administered to evaluate the therapeutic potential. Tumour growth was monitored by calliper measurements of the length (*L*) and width (*W*) and volumes calculated using the formula *V* = *L* × *W* ^2^/2. All animal experimental manipulations were approved by the Ethics Committee for Animal Experiment of Chongqing Medical University.

### 2.12 Immunohistochemistry staining

Subcutaneous transplantation tumours in mice were subjected to immunohistochemistry (IHC) staining using an antigen retrieval method with microwaves. Antibodies against phospho‐Smad3 (p‐Smad3) (Rockland, USA, catalogue 600‐401‐919) and PLK1 (Proteintech, China, catalogue 10305‐1‐AP) were utilized. The antibodies mentioned above were diluted at 1:100. Following that, the sections were washed with PBS, treated with the secondary antibody and visualized using diaminobenzidine. All sections were imaged using a Virtual Slide Microscope (VS120, Olympus, Japan). Staining intensity was assessed in a double‐blind manner using ImageJ v1.8.0 software.

### 2.13 Statistical analyses

Data are expressed as mean ± standard deviation (SD). Statistical analyses were conducted using one‐way analysis of variance (ANOVA) on GraphPad Prism 7.0 (GraphPad Software, La Jolla, CA, USA). A *p* < 0.05 was considered statistically significant.

## 3 RESULTS

### 3.1 hfCas13d specifically silences PLK1 in osteosarcoma cells

**FIGURE 1:**
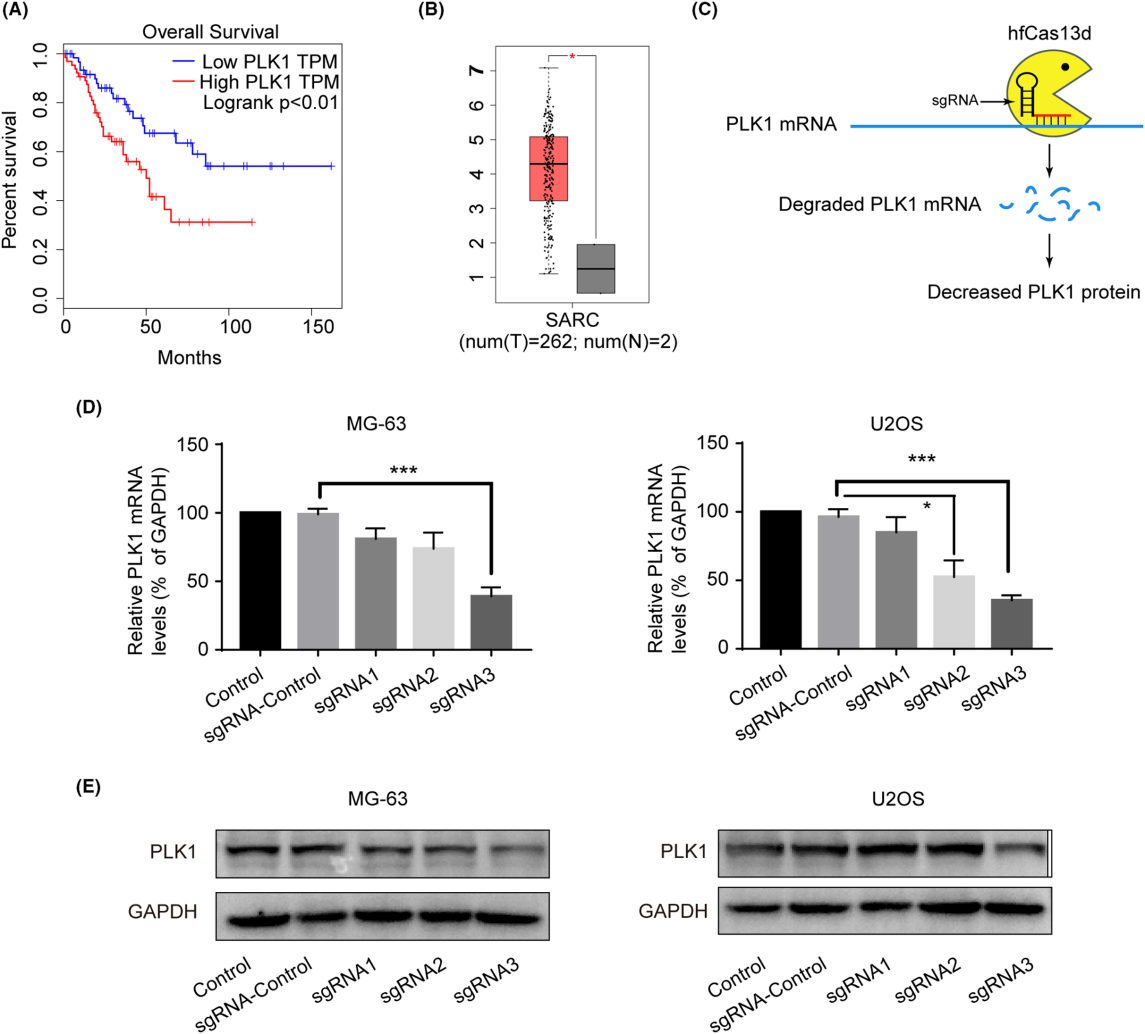
PLK1 is upregulated in osteosarcoma and efficiently knocked down by hfCas13d. (A) Kaplan‐Meier plots of overall and disease‐free survival based on PLK1 expression in osteosarcoma patients using GEPIA database. (B) PLK1 mRNA levels in osteosarcoma tumour tissues compared to normal bone tissues based on GEPIA database. (C) Schematic of lentiviral vector encoding hfCas13d and sgRNAs targeting PLK1. (D) qRT‐PCR analysis of PLK1 knockdown efficiency by different sgRNAs in MG‐63 and U2OS cells. (E) Western blot validation of PLK1 protein levels following transduction with lentiviral vectors encoding hfCas13d and sgRNAs targeting PLK1 or non‐targeting sgRNA control. All experiments were repeated three times and data represent the mean ± SD (*n* = 3 per group). *p < 0.05, ***p < 0.001 vs. sgRNA‐control.

To investigate the clinical relevance of PLK1 in osteosarcoma, we analysed PLK1 expression using the GEPIA database. PLK1 was significantly upregulated in osteosarcoma tissues compared to normal controls (Figure 1B). Higher PLK1 expression correlated with worse clinical outcomes, as evidenced by reduced overall and recurrence‐free survival rates in Kaplan–Meier analyses (Figure 1A). Next, we aimed to apply the hfCas13d system to knock down the expression of PLK1 in PDAC osteosarcoma cells (Figure 1C). To specifically target the PLK1, three gRNAs with the spacer (30 nt) were selected as candidates. Lentiviral vectors encoding hfCas13d and PLK1‐targeting sgRNAs were transduced into MG‐63 and U2OS osteosarcoma cells. Stable cells were subsequently isolated by puromycin selection. Evaluation of knockdown efficiency revealed that sgRNA2 and sgRNA3 significantly suppressed PLK1 mRNA levels, with sgRNA3 demonstrating >70% knockdown in both cell lines (Figure 1D). This was further validated at the protein level by immunoblotting, confirming sgRNA3 as the lead sgRNA construct (Figure 1E). For all subsequent experiments, cells stably expressing hfCas13d and the optimal sgRNA3 were utilized to investigate PLK1 loss‐of‐function effects.

### 3.2 PLK1 knockdown reduces osteosarcoma cellular proliferation

**FIGURE 2:**
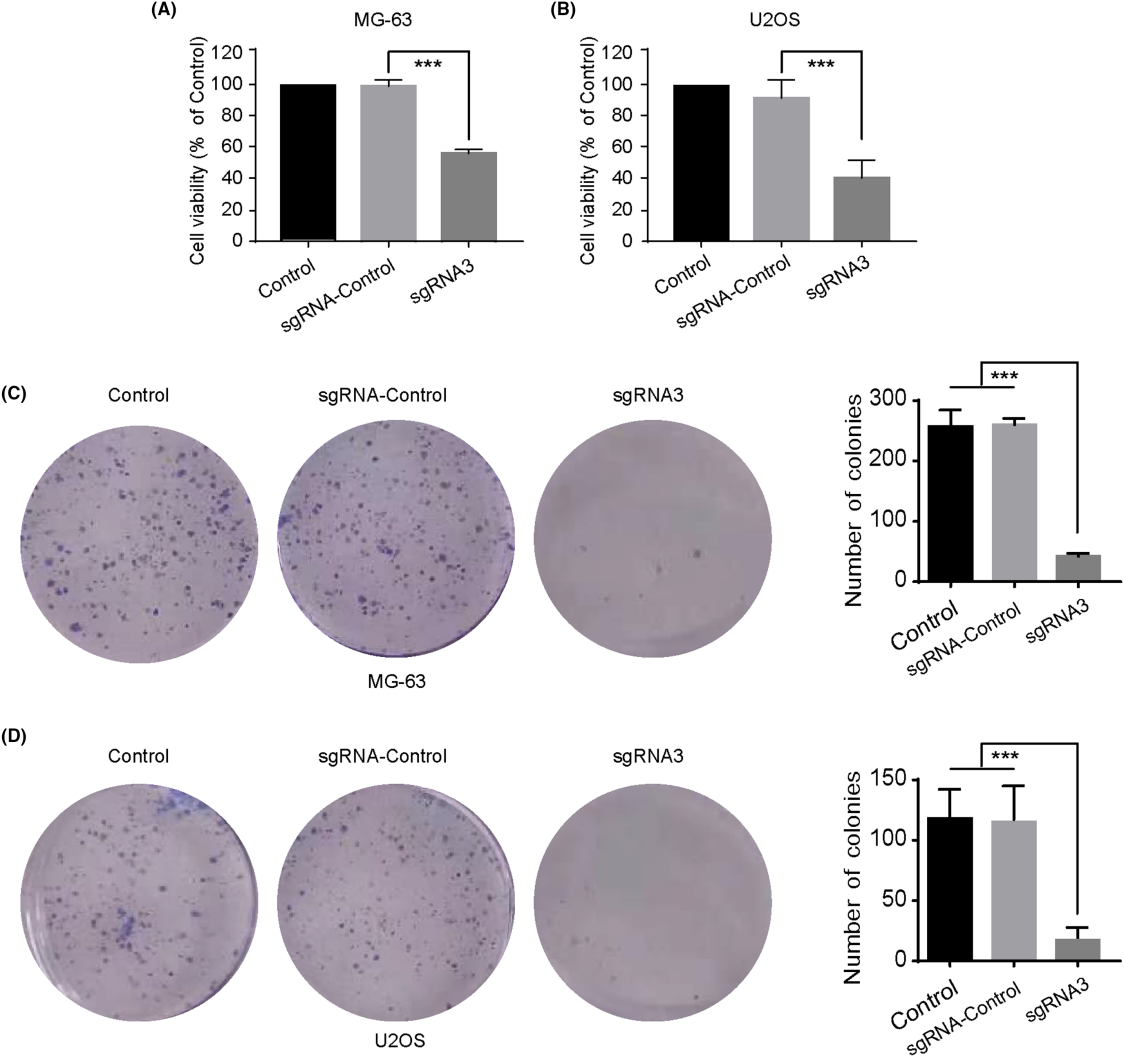
PLK1 knockdown reduces osteosarcoma cellular proliferation. Cell viability of (A) MG‐63 and (B) U2OS cells determined by CCK‐8 assay following PLK1 knockdown. Colony formation assay showing clonogenicity of (C) MG‐63 and (D) U2OS cells after PLK1 silencing. All experiments were repeated three times and data represent the mean ± SD (*n* = 3 per group). ***p < 0.001 vs. sgRNA‐control.

We next evaluated the effects of PLK1 knockdown on osteosarcoma cell viability and colony formation ability. As shown in Figure 2A, PLK1 suppression significantly reduced viability of MG‐63 cells, while the non‐targeting sgRNA‐control had no effect when compared with the control group. A similar phenomenon was observed in U2OS cells (Figure 2B). Furthermore, PLK1 silencing markedly impaired the clonogenicity of both MG‐63 and U2OS cells, as evidenced by the reduced number and size of colonies compared to controls (Figure 2C,D). In summary, the results demonstrate that targeted PLK1 suppression mediated by hfCas13d substantially reduces osteosarcoma cellular viability and proliferative capacity.

### 3.3 Silencing PLK1 arrested cell cycle of osteosarcoma cells in the G2/M phase

**FIGURE 3:**
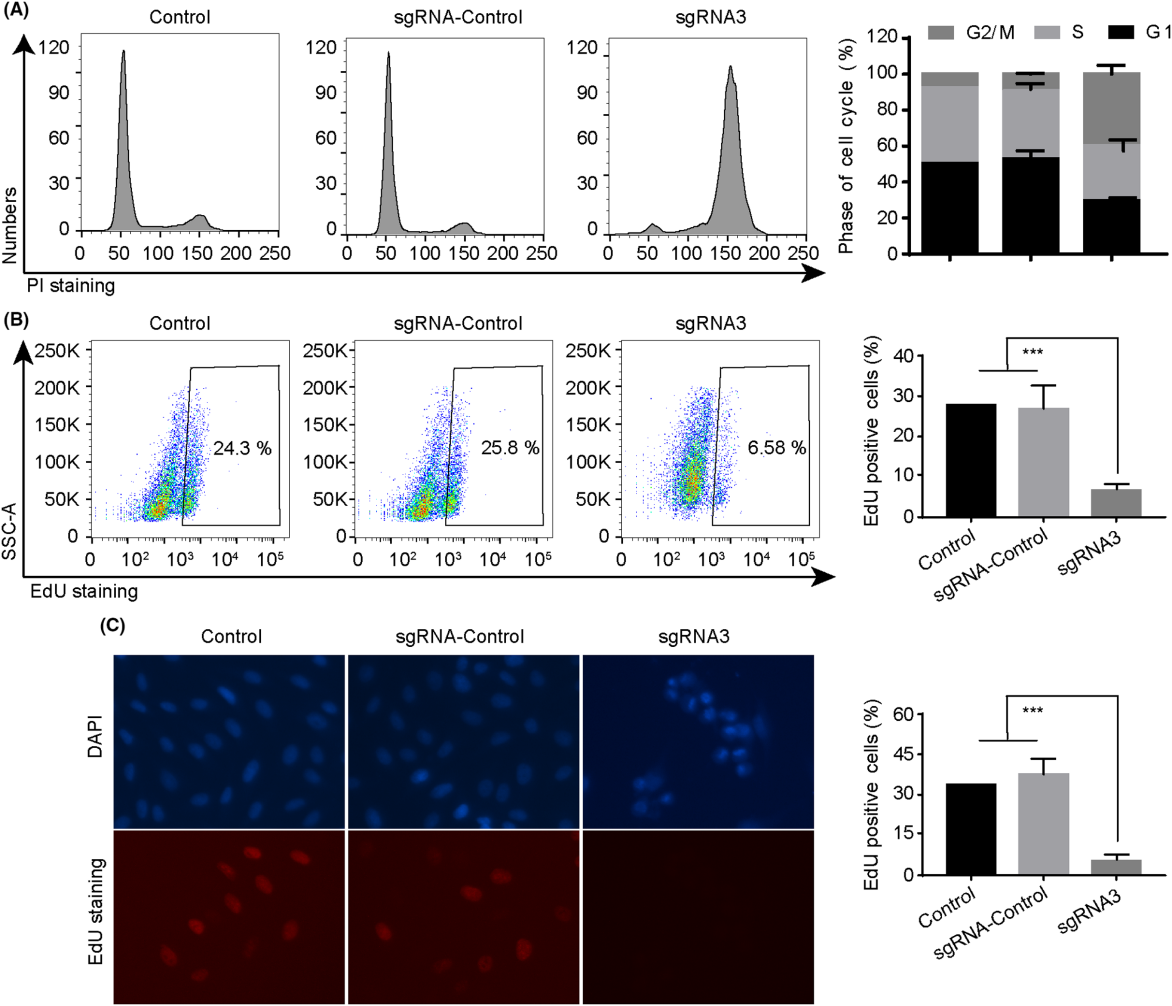
PLK1 knockdown induces G2/M arrest in MG‐63 cells. (A) Cell cycle distribution analysed by flow cytometry after PI staining. (B) DNA replication assessed by EdU incorporation assay. (C) Immunofluorescence imaging showing EdU (red) and Hoechst (blue) staining. All experiments were repeated three times and data represent the mean ± SD (*n* = 3 per group). *p < 0.05, ***p < 0.001 vs. control or sgRNA‐control.

**FIGURE 4:**
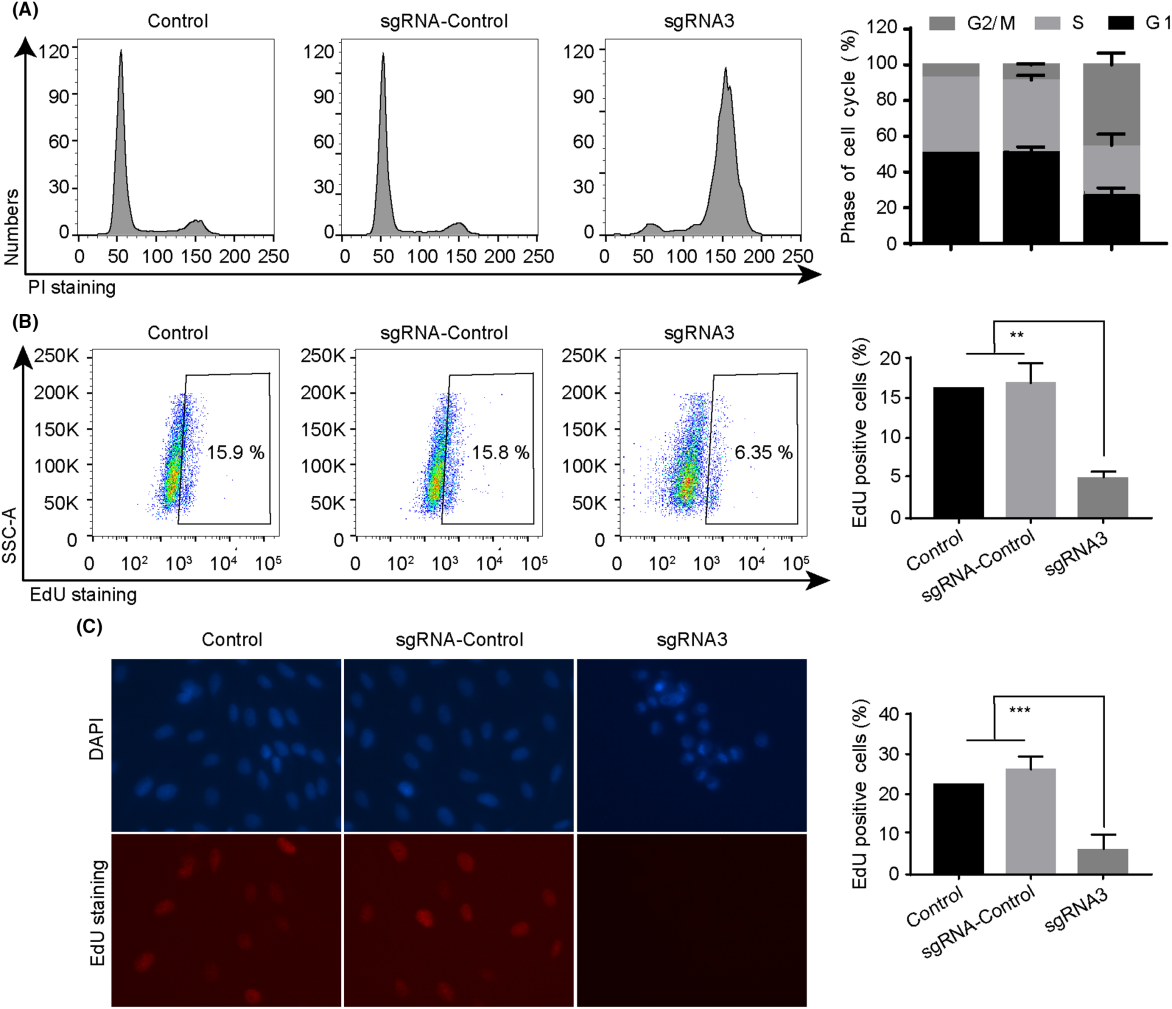
PLK1 knockdown induces G2/M arrest in U2OS cells. (A) Cell cycle distribution analysed by flow cytometry after PI staining. (B) DNA replication assessed by EdU incorporation assay. (C) Immunofluorescence imaging showing EdU (red) and Hoechst (blue) staining. All experiments were repeated three times and data represent the mean ± SD (*n* = 3 per group). ***p < 0.001 vs. control or sgRNA‐control.

Building upon this foundation, we assessed the impact of PLK1 knockdown on the cell cycle. As discerned from the outcomes presented in Figure 3A, the reduction of PLK1 in MG‐63 cells led to a decrease in the proportion of cells in the S phase, resulting in cell arrest within the G1 and G2/M phases. To further elucidate this phenomenon, we conducted an EdU incorporation assay to quantify DNA replication, particularly marking cells traversing the S phase (Figure 3B,C). Our analyses, encompassing both flow cytometry and microscopic evaluation, unequivocally demonstrated that the depletion of PLK1 markedly diminished the fraction of EdU‐positive cells. Moreover, concurrent observations via Hochest staining revealed discernible chromatin condensation in the PLK1 knockdown group, strongly suggesting that the downregulation of PLK1 may also incite apoptotic cascades. Similar results were also observed in U2OS osteosarcoma cells (Figure 4). In summary, drawing from the aforementioned findings, we proposed that the suppression of PLK1 induces cell cycle arrest in osteosarcoma cells.

### 3.4 Apoptosis induction in osteosarcoma cells by PLK1 knockdown

**FIGURE 5:**
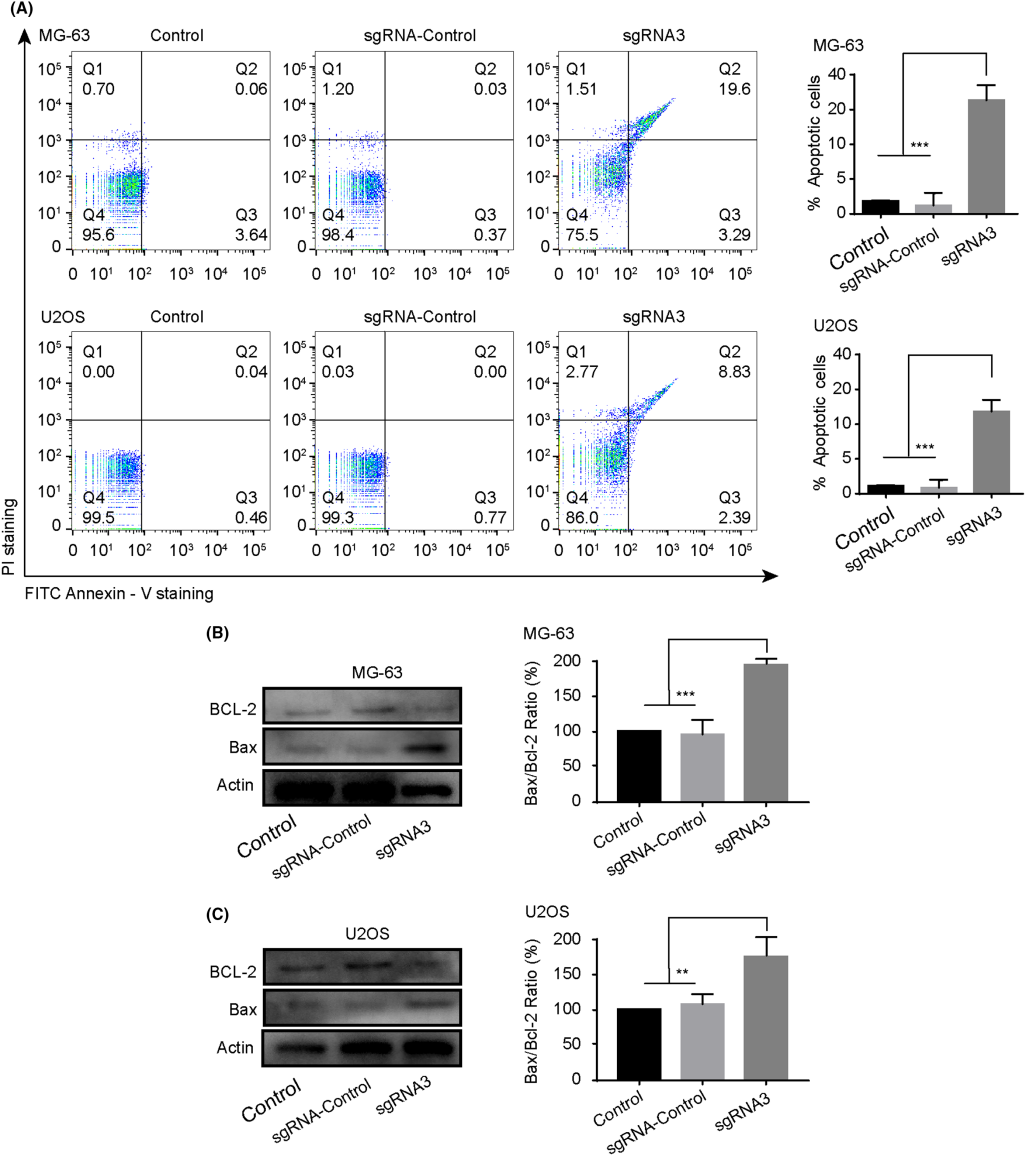
PLK1 knockdown induces apoptosis in osteosarcoma cells. (A) Apoptosis analysed by flow cytometry using Annexin V/PI staining in MG‐63 and U2OS cells. (B) Western blot analysis of pro‐apoptotic protein Bax and anti‐apoptotic protein Bcl‐2 expression. All experiments were repeated three times and data represent the mean ± SD (*n* = 3 per group). *p < 0.05, ***p < 0.001 vs. control or sgRNA‐control.

We next investigated the effects of PLK1 knockdown on osteosarcoma apoptosis. As depicted in Figure 5A, PLK1 silencing markedly induced apoptosis, as evidenced by the increased percentage of apoptotic cells in both MG‐63 (19.6%) and U2OS (8.83%) compared to controls. We further evaluated expression changes in apoptotic regulators. As shown in Figure 5B, PLK1 suppression led to upregulation of the pro‐apoptotic protein Bax concomitant with downregulation of the anti‐apoptotic protein Bcl‐2, effectively increasing the Bax/Bcl‐2 ratio to promote apoptosis. In summary, targeted PLK1 inhibition triggers intrinsic apoptotic pathways in osteosarcoma cells.

### 3.5 Silencing PLK1 blocked the invasion of osteosarcoma cells

**FIGURE 6:**
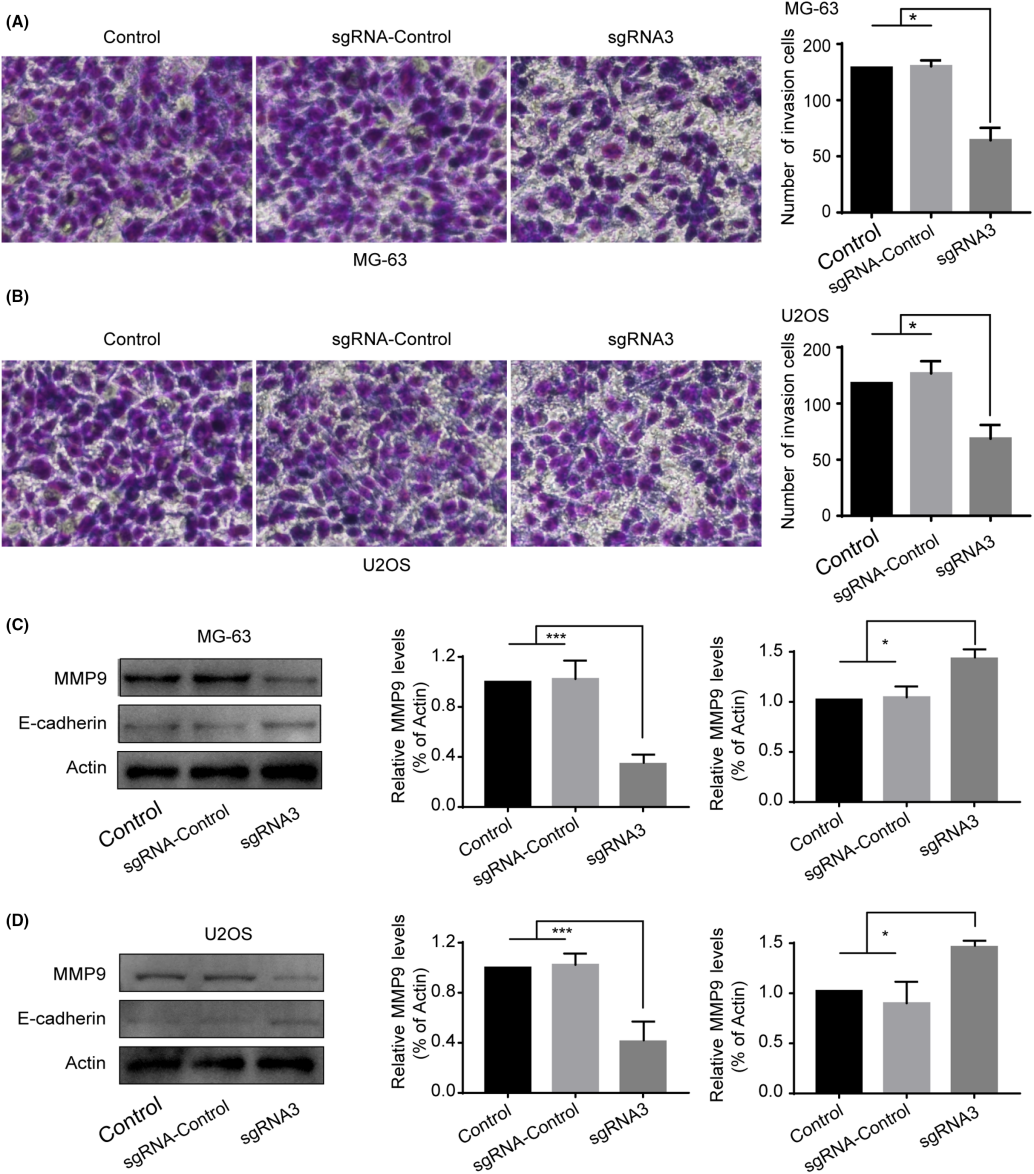
PLK1 knockdown reduces invasion of osteosarcoma cells. Transwell invasion assay showing effects on invasiveness of (A) MG‐63 and (B) U2OS cells after PLK1 silencing. (C–D) Western blot and qRT‐PCR analysis of MMP2 and E‐cadherin protein and mRNA levels in MG‐63 and U2OS cells. All experiments were repeated three times and data represent the mean ± SD (*n* = 3 per group). *p < 0.05, ***p < 0.001 vs. control or sgRNA‐control.

We next evaluated the effects of PLK1 knockdown on the invasive capacity of osteosarcoma cells. As shown in Figure 6A,B, PLK1 silencing markedly suppressed the invasiveness of both MG‐63 and U2OS cells compared to controls. We further examined expression changes in invasion‐related proteins. PLK1 knockdown significantly reduced matrix metalloproteinase‐2 (MMP2) mRNA and protein levels in osteosarcoma cells (Figure 6C,D). Interestingly, we also observed increased transcript and protein levels of the epithelial marker E‐cadherin following PLK1 suppression (Figure 6C,D). Taken together, these data suggest PLK1 silencing attenuates the invasive phenotype of osteosarcoma cells, likely by restoring epithelial characteristics.

### 3.6 Effects of PLK1 silencing on TGF‐β/Smad3 signalling in osteosarcoma

**FIGURE 7:**
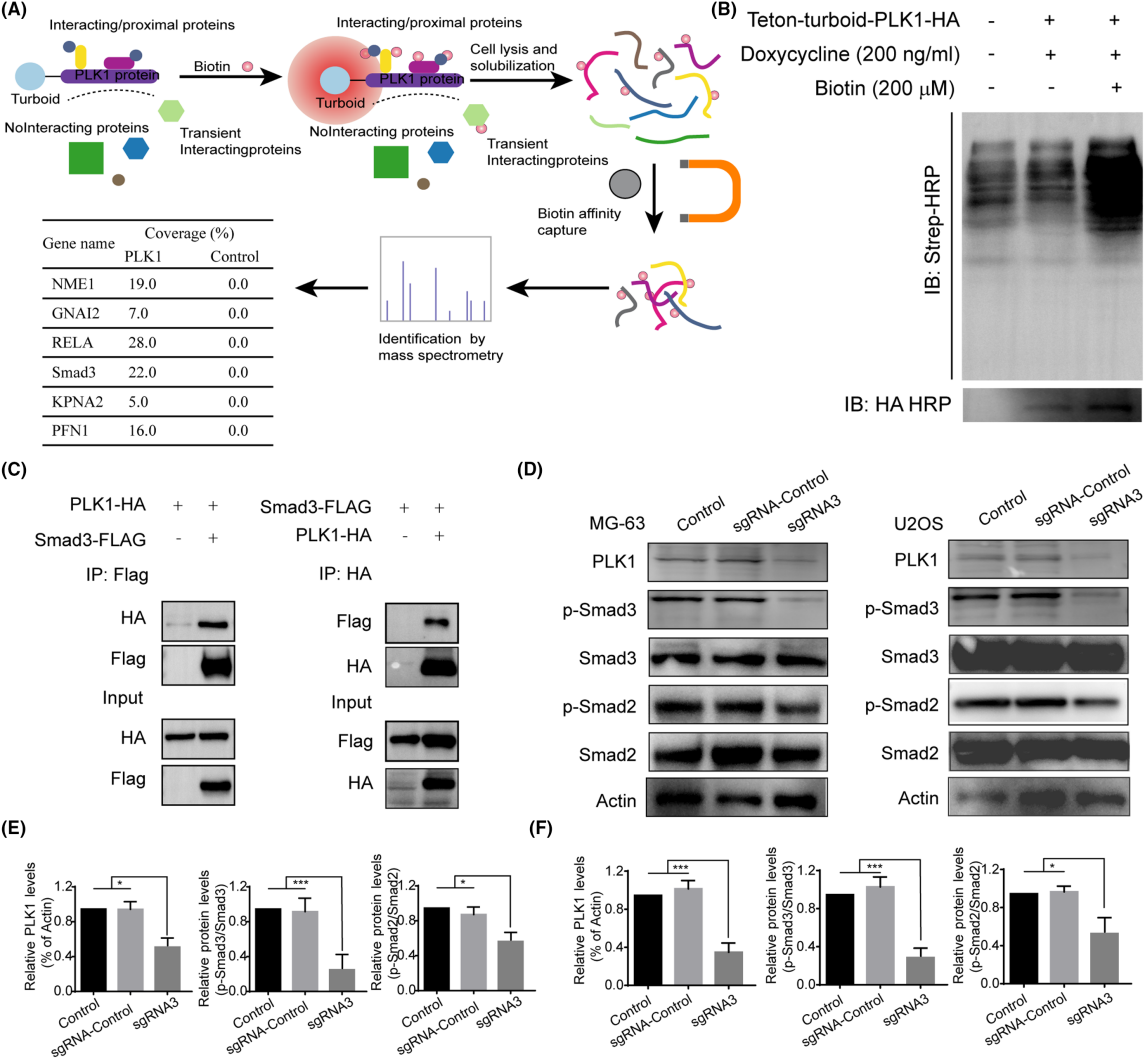
Effects of PLK1 silencing on TGF‐β/Smad3 signalling. (A) Schematic overview of proximity labelling workflow using TurboID‐PLK1. (B) Validation of biotin‐labelled PLK1 proximity interactors by streptavidin‐HRP detection. (C) Co‐immunoprecipitation confirming physical interaction between PLK1 and Smad3. (D) Western blot analysis showing Smad2/3 phosphorylation following PLK1 knockdown. All experiments were repeated three times and data represent the mean ± SD (*n* = 3 per group). *p < 0.05, ***p < 0.001 vs. control or sgRNA‐control.

To elucidate the potential PPI network of PLK1, we next performed proximity labelling method by TurboID which combines the efficient kinetics of APEX2 with the non‐toxicity of BioID coupled with mass spectrometry (MS) analysis. A schematic overview of the proximity labelling workflow is outlined in Figure 7A. Successful biotinylation of PLK1 proximity interactors was validated by streptavidin‐HRP detection, as shown in Figure 7B. MS analysis of the labelled protein eluates identified several potential PLK1‐interacting partners, including NME1, GNAI2, RELA and Smad3, and these potential interactions are illustrated in Figure 7A and detailed further in Data S1. Although Smad3 did not exhibit the highest peptide coverage in the proteomics data, we pursued it as a candidate mediator of PLK1 effects based on the PLK1 knockdown‐induced changes in MMP2 expression and the known role of Smad3 as an MMP2 transcriptional regulator.^20^ Co‐IP experiments confirmed that PLK1 physically interacts with Smad3 irrespective of which protein served as the bait in the Co‐IP assay (Figure 7C).

We next investigated the effects of PLK1 knockdown on key proteins involved in TGF‐β/Smad3 signalling. Interestingly, PLK1 silencing markedly increased Smad2 and Smad3 phosphorylation in both MG‐63 and U2OS osteosarcoma cell lines (Figure 7D,E). Taken together, these data imply that the anti‐fibrotic effects of PLK1 inhibition may be mediated through its physical interaction with Smad3 and consequent modulation of TGF‐β/Smad3 signalling.

### 3.7 PLK1 knockdown impairs osteosarcoma xenograft growth in vivo

**FIGURE 8:**
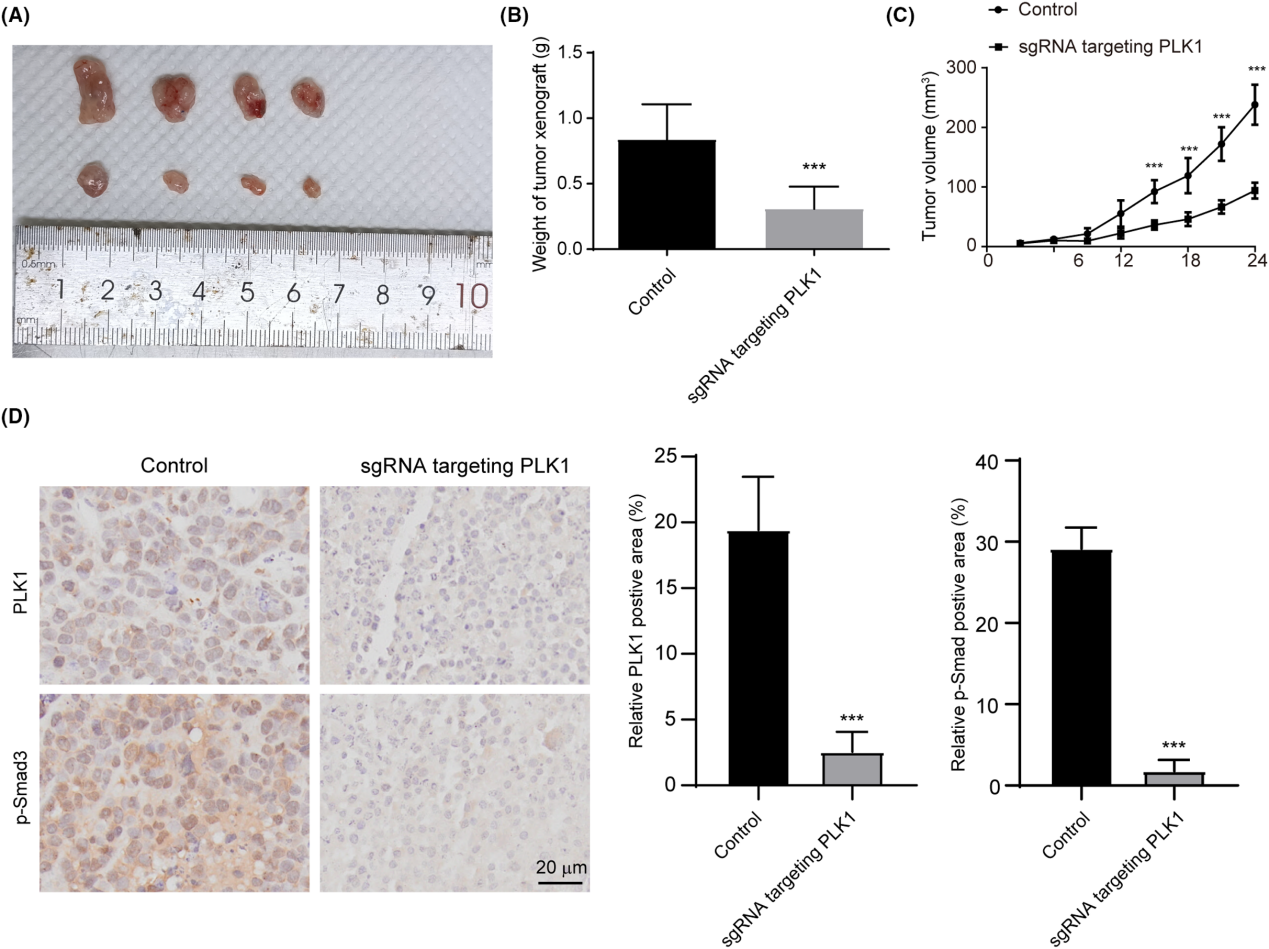
PLK1 knockdown suppresses osteosarcoma xenograft growth in vivo. (A) Tumour volumes measured after intratumoral injection of lentiviral vectors encoding hfCas13d and sgRNA targeting PLK1 or non‐targeting sgRNA control. (B) Images of dissected tumours at endpoint. (C) Tumour volume was monitored at the indicated time. (D) PLK1 and p‐Smad3 deposition measured by immunohistochemistry staining. Data represent the mean ± SD (*n* = 4 per group). *p < 0.05, **p < 0.01 vs. sgRNA‐control.

To validate the anti‐tumour effects of PLK1 suppression in vivo, we utilized a subcutaneous xenograft nude mouse model with intratumoral delivery of lentiviral vectors encoding PLK1‐targeting sgRNAs. As predicted, mice treated with PLK1 knockdown vectors exhibited significantly reduced tumour growth compared with control mice receiving empty vectors (Figure 8A–C). The IHC analysis demonstrated that xenografts in the group with hfCas13d‐mediated PLK1 suppression exhibited reduced expression of both PLK1 and p‐Smad3 compared to the control group (Figure 8D). In summary, these findings demonstrate that hfCas13d‐mediated PLK1 suppression is capable of substantially attenuating the in vivo tumour growth of osteosarcoma xenografts, potentially through the regulation of Smad3 activity.

## 4 DISCUSSION

In this study, we demonstrated that PLK1 is upregulated in osteosarcoma tissues and its overexpression correlates with poorer clinical outcomes. Using the CRISPR‐hfCas13d system, we achieved robust and specific knockdown of PLK1 in two osteosarcoma cell lines, MG‐63 and U2OS. PLK1 suppression inhibited cellular proliferation, as evidenced by reduced viability and clonogenicity. Cell cycle analysis revealed G2/M arrest, which was further supported by decreased EdU incorporation indicating reduced DNA replication. PLK1 knockdown also induced apoptosis, indicated by increased Annexin‐V staining and upregulation of the pro‐apoptotic protein Bax with concurrent downregulation of the anti‐apoptotic protein Bcl‐2. In addition, we found that PLK1 silencing reduced the invasive capacity of osteosarcoma cells, likely by restoring epithelial characteristics as shown by increased E‐cadherin expression. Through proximity labelling with TurboID coupled to MS, we identified novel interactions between PLK1 and Smad3. Follow‐up co‐immunoprecipitation experiments validated this physical association between PLK1 and Smad3. In addition, PLK1 knockdown enhanced Smad2/3 phosphorylation, suggesting its impact on TGF‐β/Smad3 signalling. More importantly, in vivo delivery of lentiviral vectors targeting PLK1 substantially reduced osteosarcoma xenograft growth in a subcutaneous nude mouse model.

Recent advancements in synthetic RNA‐based RNA interference (RNAi) techniques facilitate gene expression modulation at both translational and transcriptional levels.^21^ Despite its efficacy, RNAi's functionality is organism‐specific and prone to off‐target effects.^22^ To circumvent these limitations, researchers have adapted inactive RNA‐guided Cas9 and Cas12 variants for CRISPR interference (CRISPRi).^23^ These adaptations utilize protospacer adjacent motifs (PAMs) for enhanced target specificity. However, it is important to note that DNA‐targeting CRISPRi systems can inadvertently silence adjacent genes within operons.^24^ In contrast, RNA‐targeting CRISPR‐Cas systems, particularly the Cas13 protein family, have emerged as effective tools for transcriptome engineering, with broad applications in research and therapeutics.^14^, ^17^, ^25^, ^26^, ^27^, ^28^ The Cas13 proteins, guided by a single RNA molecule (gRNA), exhibit high specificity and efficiency in targeting and cleaving specific RNA transcripts.^29^ Among these, RfxCas13d, or CasRx, an orthologue of CRISPR–Cas13d, has shown superior RNA knockdown efficiency and specificity compared to Cas13a and Cas13b variants. Furthermore, while HfCas13d has been engineered for enhanced accuracy and exhibits no detectable off‐target effects, it is important to acknowledge its limitations.^16^ Notably, despite its genetic optimization, HfCas13d has a substantial molecular size with 967 amino acids, resulting in a relatively large molecular weight.^16^ This significant size can pose challenges in certain applications, particularly in terms of delivery efficiency and cellular uptake. Our findings highlight the oncogenic functions of PLK1 in promoting osteosarcoma progression and suggest PLK1 is a promising therapeutic target in this disease. Although small molecule PLK1 inhibitors have been developed, our study demonstrates the utility of CRISPR‐Cas13d for targeted PLK1 suppression without detectable off‐target effects. Further optimization of delivery methods could enhance the clinical potential of this approach. Cas13d‐mediated PLK1 knockdown could be combined with nanoparticle delivery or engineered extracellular vesicle to enable systemic administration.^30^

The novel PLK1–Smad3 interaction we identified provides mechanistic insight into how PLK1 knockdown impacts TGF‐β/Smad3 signalling and downstream targets like MMP2.^20^ In addition, a previous report has also demonstrated that PLK1 could provoke phosphorylation of vimentin, by recruiting Smad2/3 to PD‐L1 promoter.^9^ The physical association between PLK1 and Smad3 likely enables PLK1 to directly modulate Smad3 activity through phosphorylation at specific residues. It will be important for future studies to precisely map the PLK1‐mediated phosphorylation sites on Smad3 and elucidate how phosphorylation affects Smad3 function and transcriptional activity. This could uncover new ways in which PLK1 promotes disease progression.

Our work has some limitations. While we demonstrated anti‐tumour effects in a subcutaneous xenograft model, testing in orthotopic and metastatic models will better recapitulate osteosarcoma progression. It will also be necessary to thoroughly evaluate potential toxicities associated with systemic PLK1 suppression. Further research could explore synergistic combinatorial therapies, such as pairing PLK1 inhibitors with standard chemotherapy. Additional work is also needed to translate this therapeutic strategy into human clinical trials. Overall, this study establishes a foundation for additional investigation into targeted PLK1 inhibition hfCas13d‐mediated gene knockdown as a novel therapeutic approach in osteosarcoma.

## AUTHOR CONTRIBUTIONS

**Yi Yuan:** Conceptualization (lead); data curation (lead); methodology (lead); writing – original draft (lead). **Daigui Cao:** Conceptualization (equal); data curation (equal); formal analysis (equal); validation (equal). **Anwei Zhang:** Conceptualization (supporting); data curation (supporting). **Zhiwei Liu:** Data curation (supporting); formal analysis (supporting); validation (equal); writing – original draft (supporting). **Zhongliang Deng:** Project administration (equal); supervision (equal); writing – review and editing (equal). **Shengli Zhang:** Project administration (equal); supervision (equal).

## FUNDING INFORMATION

This study was supported by Key Research and Development Plan for Science and Technology Projects in Dazhou City (No. 22ZDY0050) and Natural Science Foundation in Chongqing City (No. cstc2021jcyj‐msxmX1040).

## CONFLICT OF INTEREST STATEMENT

The authors declare that there are no conflicts of interest.

## Supporting information


Data S1.

## Data Availability

The data that support the findings of this study are available from the corresponding author upon reasonable request.
